# Clinical Implementation of Expanded Carrier Screening in Pregnant Women at Early Gestational Weeks: A Chinese Cohort Study

**DOI:** 10.3390/genes12040496

**Published:** 2021-03-29

**Authors:** Mengmeng Shi, Angeline Linna Liauw, Steve Tong, Yu Zheng, Tak Yeung Leung, Shuk Ching Chong, Ye Cao, Tze Kin Lau, Kwong Wai Choy, Jacqueline P. W. Chung

**Affiliations:** 1 Department of Obstetrics and Gynaecology, The Chinese University of Hong Kong, Hong Kong, China; shimengmeng@link.cuhk.edu.hk (M.S.); liauwlinna@yahoo.com.hk (A.L.L.); steve.tong@hkdnalab.com.hk (S.T.); HaleyZheng@link.cuhk.edu.hk (Y.Z.); tyleung@cuhk.edu.hk (T.Y.L.); chongsc@cuhk.edu.hk (S.C.C.); yecao@cuhk.edu.hk (Y.C.); 2 The Chinese University of Hong Kong-Baylor College of Medicine Joint Center of Medical Genetics, Hong Kong, China; 3 Department of Paediatrics, The Chinese University of Hong Kong, Hong Kong, China; 4 Hong Kong Hub of Paediatric Excellence, The Chinese University of Hong Kong, Hong Kong, China; 5 Department of Obstetrics and Gynaecology, Prince of Wales Hospital, Hong Kong, China; drtklau@hkparamount.com

**Keywords:** expanded carrier screening, Chinese, Hong Kong, recessive disorders

## Abstract

Demands for expanded carrier screening (ECS) are growing and ECS is becoming an important part of obstetrics practice and reproductive planning. The aim of this study is to evaluate the feasibility of a small-size ECS panel in clinical implementation and investigate Chinese couples’ attitudes towards ECS. An ECS panel containing 11 recessive conditions was offered to Chinese pregnant women below 16 gestational weeks. Sequential testing of their partners was recommended for women with a positive carrier status. The reproductive decision and pregnancy outcome were surveyed for at-risk couples. A total of 1321 women performed ECS successfully and the overall carrier rate was 19.23%. The estimated at-risk couple rate was 0.83%. Sequential testing was performed in less than half of male partners. Eight at-risk couples were identified and four of them performed prenatal diagnosis. Our study demonstrated that a small-size ECS panel could yield comparable clinical value to a larger-size panel when the carrier rate of the individual condition is equal or greater than 1%. In addition, more than half of male partners whose wives were carriers declined any types of sequential testing possibly due to a lack of awareness and knowledge of genetic disorders. Genetic education is warranted for the better implementation of ECS.

## 1. Introduction

Autosomal recessive (AR) and X-linked disorders affect an estimated 1 in 300 pregnancies worldwide [1]. These disorders can lead to intrauterine death, neonatal death, or chronic disability, and account for approximately 20% of infant mortality and 10% of infant hospitalizations [2]. Since most of the carriers of AR and X-linked disorders are asymptomatic, couples lack awareness about their carrier status. Carrier screening enables couples to realize their risk of having a child with recessive conditions and enables them to consider alternative reproductive options [3]. Carrier screening tests have historically been implemented to assess a relatively small number of conditions characterized by high frequency and motility rates in certain ethnic groups [4,5,6]. In recent years, the rapid development of next-generation sequencing (NGS) has allowed the simultaneous screening of dozens or hundreds of disorders, regardless of ethnicity, in a single assay [7]. This panel-based screening is known as expanded carrier screening (ECS). The American College of Obstetricians and Gynecologists (ACOG) recently has recognized ECS as an acceptable strategy for preconception and prenatal screening [8]. 

Despite growing numbers of ECS studies ranging from panel evaluation to clinical practice, most of them are focused on reporting prevalence especially among western populations [9,10,11]. Data regarding the implementation of ECS in the Chinese population is still limited. Lazarin et al. investigated carrier status for around 400 disease-causing variants in around 20,000 people from different ethnic groups and reported the lowest carrier rate (8.5%) in the East Asian population [10]. However, emerging studies recently have suggested that carrier rates of recessive conditions in the Chinese population may be underestimated. Zhao et al, screened 11 Mendelian disorders in 10,476 prenatal/preconception couples of the southern China, and the overall carrier rate was unexpectedly high (27.49%) [12]. The striking difference between these two studies may owe to the fact that the ECS panel used by Lazarin et al. was designed based on Caucasian or Ashkenazi Jewish populations.

In Hong Kong, thalassemia screening has been offered at preconception check-ups or first antenatal checks due to its high prevalence [13,14]. Mean cell volume (MCV) and mean corpuscular hemoglobin (MCH) screening were performed to identify the suspected thalassemia traits in patients [13]. With this approach, the incidence of newborn thalassemia major or hydrops cases has dropped dramatically, which greatly lessens the economic burden on the health care system [14]. However, this routine screening could not identify silent alpha-thalassemia carriers who only have a loss of one copy of the alpha-thalassemia gene. These individuals are clinically and hematologically normal and so can only be detected by the genetic testing [15]. In addition, there is a lack of genetic testing clinically performed to screen for serious recessive conditions including thalassemia parallelly in Hong Kong. Herein, we offered an ECS panel containing 11 conditions to Chinese Hong Kong women at early gestational weeks. We aim to evaluate the feasibility of this small-size ECS panel in clinical implementation and investigate the attitude towards ECS of local couples.

## 2. Materials and Methods

### 2.1. Study Population and Clinical Practice

**Figure 1:**
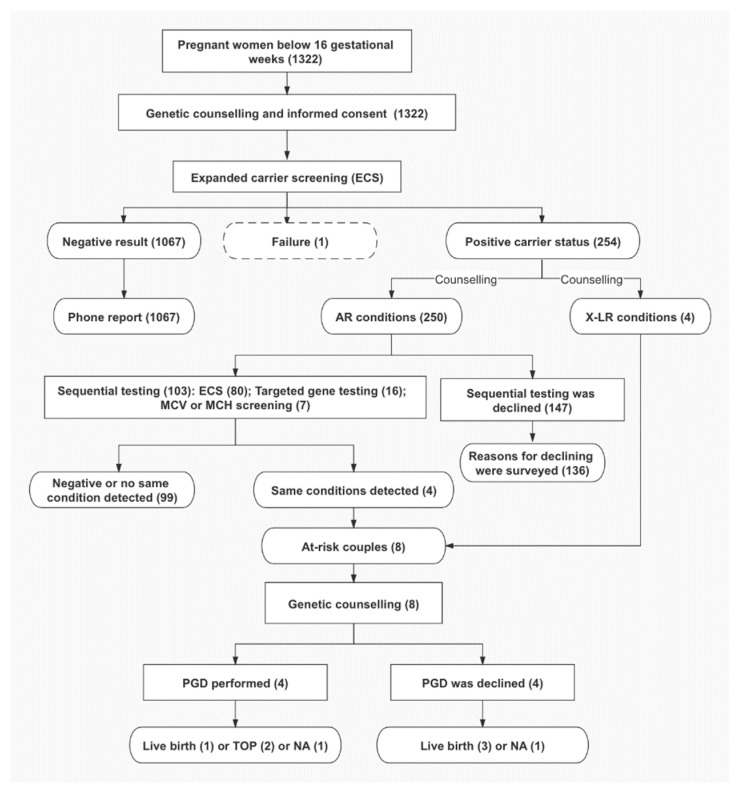
Clinical workflow of this study. Numbers of subjects are indicated in brackets. AR: autosomal recessive; X-LR: X-linked recessive; MCV: mean cell volume; MCH: mean corpuscular hemoglobin; PGD: prenatal genetic diagnosis; TOP: termination of pregnancy; NA: not available due to loss of follow up.

The study protocol was approved by the Ethics Committee of the Joint Chinese University of Hong Kong—New Territories East Cluster Clinical Research Ethics Committee (CREC Ref. No. 2019.148). This was a single center study conducted in a specialized Maternal Fetal Medicine Clinic in Hong Kong from March 2017 to September 2018. This ECS panel containing 11 recessive conditions was offered to pregnant women having non-invasive prenatal tests (NIPTs) below 16 gestational weeks. Medical records of these pregnant women accepting ECS were retrieved. Three mL of peripheral EDTA blood was obtained with written and signed consent from each pregnant woman. For the individuals with a negative carrier status, a phone report was received, while for those with a positive carrier status genetic counselling was offered and self-financed sequential screening of the male partners was recommended. If both couples were carriers of the same autosomal recessive diseases or the pregnant women was a carrier of any X-linked diseases, the risk of having an affected child was counselled. The option of prenatal genetic diagnosis was offered to determine the genetic status of the fetus. If the couple had a severely affected fetus and wanted to have termination of pregnancy this can be done legally before 24 weeks of gestation in Hong Kong. The clinical workflow of this study is illustrated in the Figure 1. 

### 2.2. Panel Design

The selection of the recessive diseases screened was based on our previous study [16]. We also followed the criteria recommended by the American College of Medical Genetics (ACMG) and ACOG [8,17] for common genetic conditions in locality, including conditions with a carrier frequency of 1 in 100 or greater; with a detrimental effect on quality of life; causing cognitive or physical impairment; requiring surgical or medical intervention; with an early onset. Eight AR conditions and three X-linked recessive conditions were selected, including dystrophinopathies (DMD, Duchenne muscular dystrophy, OMIM: #310200 & BMD; Becker muscular dystrophy, OMIM: #300376); alpha-thalassemia (OMIM: #604131); beta-thalassemia (OMIM: #613985); autosomal recessive deafness 1A (DFNB1, OMIM: #220290); autosomal recessive deafness 4 with enlarged vestibular aqueduct (DFNB4, OMIM: #600791); phenylketonuria (PKU, OMIM: #261600); glycogen storage disease type II (OMIM: #232300); Wilson’s disease (OMIM: #277900); hemophilia A (OMIM: #306700); hemophilia B (OMIM: #306900); and spinal muscular atrophy (SMA, OMIM: #253300). The severity of these diseases was classified as moderate, severe, or profound according to the algorithm developed by Lazarin et al. [18].

### 2.3. Next-Generation Sequencing and Complementary Gene Tests

Genomic DNA was extracted from peripheral blood samples using DNeasy Blood & Tissue Kits (QIAGEN). Sequence-enrichment DNA probes targeting the 12 genes were designed and commercially obtained from the NimbleDesign system (NimbleGen, Roche). The DNA probes included all coding exons along with their 30-bp flanking intronic regions. Targeted exon captures were performed according to the standard protocol. Next-generation sequencing was then performed on the BGI-SEQ500 platform (PE 50). A Burrows–Wheeler Aligner was used to map reads to the reference genome hg19, and the Genome Analysis Toolkit (Broad Institute) was used to detect single nucleotide variants (SNVs) and small indels within 10 bps. In this panel, Q-PCR was also performed for exon 7 deletion in *SMN1* gene. However, it cannot differentiate the two copies of *SMN1* on a chromosome in cis (silent carrier) or in trans based on this assay. For alpha-thalassemia, Gap-PCR was used to detect five mutations, -SEA, -α3.7, -α4.2, -FIL, and -THAI in the *HBA1/HBA2* gene. For hemophilia A, long-PCR was used in detecting the exon 1 and 22 inversions in the *F8* gene. The analytical sensitivity and specificity of this ECS panel is 99%.

### 2.4. Variant Interpretation and Data Analysis

The classification and interpretation of variants were performed according to the standards and guidelines recommended by the ACMG and published in the literature [19]. Only “pathogenic” and “likely pathogenic” variants were reported according to the recommendations [17,20]. The pathogenic variant: *GJB2* c.109G>A (p.Val37Ile) was not reported to patients in this panel due to its low penetrance [21]. Likely pathogenic variants were reported as they have a high likelihood of being disease-causing, though additional evidence is expected to confirm this assertion of pathogenicity [19]. At-risk couples were defined as couples with pathogenic or likely pathogenic variants on the same AR disorders genes, or in which the female partners had pathogenic or likely pathogenic variants for the X-linked disorders [3]. The variant carrier rate (VCR) was defined as the proportion of individuals with pathogenic and likely pathogenic variants in this cohort. Gene carrier rates (GCRs) were defined as the estimated proportion of individuals with at least one pathogenic or likely pathogenic variants in a specific gene. Cumulative carrier rates (CCRs) were defined as the estimated detection rate of the carrier screening panel. The at-risk couple rate (ACR) was defined as the probability that the couple both were carriers of pathogenic or likely pathogenic variants in the same gene. The calculations of VCR, GCRs, CCRs, and ACR were done according to the formula developed by Guo and Gregg’s study [22]. Pearson’s Chi-square and linear-by-linear association were used to compare the sequential testing compliance rates of male partners among women with different carrier statuses. *P* < 0.05 was considered statistically significant.

## 3. Results

### 3.1. Population Demographics

During the period of March 2017 to September 2018, a total of 1322 pregnant Chinese women (<16 gestational weeks) accepted ECS with 11 conditions as complimentary genetic screening when they received NIPTs in a specialized Maternal Fetal Medicine Clinic in Hong Kong. One subject in this study was excluded due to a technical problem in the laboratory testing. The mean age of the recruited pregnant women was 33.50 ± 4.05 years old and the mean gestational age on the day of the test was 12.34 ± 1.08 weeks of gestation. Most women (1185, 89.70%) conceived naturally, and only a small fraction (129, 9.77%) conceived with assisted reproductive technology including in vitro fertilization, ovulation induction, and intra-uterine insemination.

### 3.2. Disease Carrier Rates

The carrier frequencies of the 11 conditions are presented in Table 1. Among the 1321 women screened, 19.23% of individuals (*n* = 254) were carriers for at least one recessive condition. If thalassemia was removed from the panel, the carrier rate was 10.14% (*n* = 134). Among the 254 carriers, 17.71% (*n* = 234) of individuals carried one recessive condition, while 1.51% (*n* = 20) of individuals carried two different recessive conditions. No individual carried more than two disease alleles in this study. The carrier rates of eight AR conditions were all above 1%. The most common condition was alpha thalassemia, with a carrier rate of 7.80% (*n* = 103). Other conditions such as beta thalassemia, DFNB4, and PKU are also prevalent, with carrier rates of 2.27% (*n* = 30), 2.04% (*n* = 27), and 2.27% (*n* = 30), respectively. The carrier rate of hemophilia A was 0.23% (*n* = 3). The variants we identified in this cohort are shown in Table S1.

### 3.3. Estimated Yield of ECS Panel

We further estimated the detection yields of eight AR conditions in this ECS panel in 1321 Chinese women. The other three were not calculated, as the formula was not applicable for X-linked conditions. VCRs, GCRs, CCRs, and ACR were calculated and GCRs are shown in Table 2. All estimated GCRs of eight AR conditions were above 1% and the estimated CCR was 18.69%. The ACR was around 0.83%, which means that at least 1 in 120 couples would be identified as at-risk by this ECS panel. 

### 3.4. Sequential Testing of Male Partners

For female carriers, genetic counselling was provided and sequential testing of their partners was recommended for the women carrying AR conditions (*n* = 250). Among these 250 women, 147 (58.80%) of their partners declined any sequential testing, thus the sequential testing compliance rate was 41.20% (*n* = 103). The most common reason for declining was that the male partner had been checked before (46.94%, *n* = 69) for thalassemia carrier status by MCV and MCH screening, which has been routinely offered to all couples of childbearing age at every public hospital and Maternal and Child Health Centre in Hong Kong. The other reasons included the acceptance of an affected child for carriers of PKU or autosomal recessive deafness, no risk of lethal type of alpha thalassemia (Hb Bart syndrome) for silent thalassemia carriers, and high cost (Table 3). For the 103 male partners who accepted sequential testing, most of them (77.67%, *n* = 80) performed the ECS panel, 15.53% (*n* = 16) of them performed targeted gene testing (multiplex ligation-dependent probe amplification for SMA disease), and the rest (6.80%, *n* = 7) performed MCV or MCH screening for thalassemia (Figure 1). Sequential testing compliance rates of male partners were significantly higher when women carried with two conditions (Table S2) or more serious conditions (Table S3). 

### 3.5. At-Risk Couples and Results-Guided Behaviors:

There were eight at-risk couples identified in our cohort, including four with autosomal recessive diseases and four with X-linked recessive diseases (Table 4). Four couples performed prenatal diagnosis and three (75%) were identified to have affected fetuses with SMA, hemophilia A, and PKU respectively. Couples with fetuses affected by SMA and hemophilia A decided to terminate the pregnancy before 24 gestational weeks. As the homozygous *PAH* c.158G>A (p.R53H) variant can either exist in patients or normal individuals, the pathogenicity of this variant is controversial [23,24]. After counselling, the couple with the *PAH* c.158G>A (p.R53H) variant decided to continue with the pregnancy. Newborn metabolic screening was also performed after delivery, and results returned normal.

## 4. Discussion

In this study, a small-size ECS panel containing eight AR and three X-linked conditions was offered to 1321 Chinese pregnant women at early gestational ages. Nearly 20% of the women were detected to carry at least one condition. The carrier rates of all eight AR conditions were above 1% and alpha thalassemia showed the highest carrier rate (7.80%, *n* = 103). The estimated ACR of this ECS panel was 0.83%, which suggested that at least 1 in 120 couples would be at risk of having an affected child. Among the 250 women carrying AR conditions, 41.20% (*n* = 103) performed sequential testing for their male partners. In total, eight at-risk couples were identified and four of them performed prenatal diagnosis. 

### 4.1. Would a Larger-Size ECS Panel Be Better?

Determining the included conditions and capacity of an ECS panel is always an issue of hot debate. The likelihood of identifying a carrier status increases if more conditions are screened in one panel. Despite the low prevalence of each condition, the larger number of diseases being screened may result in more than half of individuals being identified as carriers. However, this definitely increases challenges for genetic counselling, medical expenses, and also the anxiety of patients [25]. The ACMG has stated that rather than including as many diseases as possible during simultaneous testing, the appropriate selection of diseases should be prioritized [26]. One of the criteria in our study is disease severity [17]. All the selected 11 conditions are either severe or profound, except for DFNB1 and DFNB4 which are considered as moderate conditions. Nevertheless, we still included these two diseases in this panel due to the high prevalence previously reported in our local population [16]. Phenylketonuria was also included though it is detectable through newborn screening and effectively prevented with a special diet. Carrier screening could serve as complementary testing for newborn screening and provide additional value. For example, carrier screening could help couples realize their carrier status which cannot be revealed by a negative result of newborn screening, and their risk of an affected baby could be estimated. This would significantly impact the counselling and clinical management. If a couple is detected to be at risk of phenylketonuria, prenatal diagnosis can be offered, and subsequent postnatal management can be carried out directly without waiting for newborn screening results. In addition, carrier rate is also an important criterion for inclusion. Xi et al. screened 201 genes for 135 recessive conditions in 2923 assisted-reproductive technology couples. If the genes with GCRs >1% were included, the estimated ACR was 0.73%, while if including other autosomal genes with the GCRs <1%, the estimated yield did not increase much and the estimated ACR was 0.86% [27]. In our small-size panel, all estimated GCRs of eight AR conditions were higher than 1% and the estimated ACR is around 0.83%, which is comparable to previously reported larger-size ECS panels. Thus, the selection of conditions with a carrier rate of 1% or greater is a useful threshold and provides a balance between generating a considerable yield and minimizing anxiety associated with identifying carriers of extremely rare disorders [8]. 

### 4.2. The Patients’ Attitudes towards ECS and Influential Factors

Currently, there is limited data characterizing the public perception of genetic carrier screening. Pereira et al. completed a survey among women of reproductive ages. Most of them felt positive or neutral towards ECS while half reported no interests in testing. If they were screened to be positive carriers, around 50% of them would not proceed with the sequential testing of their partners [28]. There are multiple factors affecting attitudes and opinions towards ECS held by patients, such as familiarity with genetic conditions, residual risk of diseases screened, stress from testing, and financial concerns [29]. Herein, we performed ECS in a sequential approach, which means that the male partners would be re-called for testing only if their pregnant partner tested positive. However, more than half of these partners declined any type of sequential testing including ECS or targeted genetic testing. The most common reason was that the male partners had done MCV or MCH screening for thalassemia before, though it was acknowledged that MCV and MCH values have been shown to be inaccurate predictors of the genotype for thalassemia [30]. A lack of awareness and knowledge of genetic disorders could be one of reasons why these couples showed a poor desire for ECS. In addition, patients in our study were more positive about undertaking sequential testing when they were aware of the severity of carrier conditions and high likelihood of affected offspring. Therefore, proper pre- and post-test counseling is also important in affecting the decision making of patients [29].

### 4.3. Issues to Consider for Counselling

Although ECS has been shown to be beneficial in reducing the prevalence of severe disability and recessive genetic disorders owing to the results-guided reproductive behavior of at-risk couples [31], there are still many limitations of ECS to consider, such as unexpected results, uncertainty about the correlation between phenotype and genotype, and extra expenses associated with the downstream testing. Therefore, thorough and personalized genetic counseling is essential in order for the proper clinical implementation of ECS for each couple. In this study, we did not report variants of uncertain significance (VUS) to patients, though some of them could be upgraded as disease-causing by future reclassification. Several clinics reported VUS only if a pathogenic or likely pathogenic variant was detected in the partner, which could increase the detection yield of at-risk couples [32]. However, the interpretation and counselling of such results is a great challenge and may induce the waste of medical resources [19]. In addition, some pathogenic or likely pathogenic variants such as *GJB2* c.109G>A (p.Val37Ile), *GJB2* c.101T>C (p.Met34Thr), and *GAA* c.-32-13T>G are associated with mild phenotypes, adult-onset, or low/inconclusive penetrance [21,33,34]. Different laboratories may take different attitudes towards reporting them. Genetic counselors or clinicians should be very cautious when explaining the results to patients, as counselling directly affects the decision making of carriers. There was a couple in our cohort carrying the *F8* c.1569G>T (p.Leu523=) pathogenic variant, which was associated with a mild type of hemophilia [35]. Possible outcomes and the option of prenatal diagnosis were explained to this couple. They could accept the baby with mild hemophilia and finally decided to keep the pregnancy without any further invasive prenatal testing. Of note, some laboratories currently provide customized options to take out variants with mild phenotypes, which enables more personalized and accurate guidance to patients.

### 4.4. Limitations and Outlook

One of the limitations for this study is that we only selected the representative conditions in the current ECS panel and these may not cover all the common recessive conditions in the locality. Based on our pilot data with a small sample size, several candidate conditions such as 21-Hydroxylase deficient congenital adrenal hyperplasia, Krabbe’s disease, and CLN5-related neuronal ceroid lipofuscinosis should be further investigated in the Chinese population [36]. In order for a well-designed ECS panel, efforts are still needed to learn about the carrier status of the Chinese population more comprehensively. Nowadays, next generation sequencing (NGS) is unveiling a large number of variants of controversial significance, such as the HFE c.187C>G and CFTR 5T variants [37,38]. These variants are usually associated with variable presentations and may not be appropriate for general population screening. Experimental evidence and updated population data may help to solidify genotype–phenotype correlations. Currently, there is no dedicated guidance available for how best to approach them. More study and practice are warranted to learn about how these variants affect medical management and patient decision making.

## 5. Conclusions

In this study, we preliminarily investigated the carrier status of Chinese pregnant women at early gestational weeks and explored the feasibility of a small-size ECS panel as well as the Chinese couples’ attitudes towards ECS. ECS provided comprehensive information about the carrier status of patients and reduced the risk of genetic disorders in their offspring. The small-size ECS panel also showed comparable estimated yields to the larger-size panel, but required the cautious selection of diseases screened. In addition, more than half of the patients’ partners were still reluctant to perform sequential testing possibly due to a lack of knowledge about genetic diseases. In order to ensure the successful implementation of ECS, efforts should also be made to create awareness and improve genetic education for the public. 

## Figures and Tables

**Table 1:** Carrier rates of 11 conditions in pregnant women at early gestational weeks.

Diseases	Inheritance Mode	Classification	Genes	No. of Cases (n)	Carrier Rate	Carrier Rate (1 in)
Alpha thalassemia	AR	Severe or profound	*HBA1/HBA2*	103	7.80%	12.8
Beta thalassemia	AR	Severe	*HBB*	30	2.27%	44.0
Autosomal recessive deafness 1A	AR	Moderate	*GJB2*	22	1.67%	60.0
Autosomal recessive deafness 4 with enlarged vestibular aqueduct	AR	Moderate	*SLC26A4*	27	2.04%	48.9
Phenylketonuria	AR	Severe	*PAH*	30	2.27%	44.0
Wilson’s Disease	AR	Severe	*ATP7B*	22	1.67%	60.0
Spinal muscular atrophy	AR	Profound	*SMN1*	21	1.59%	62.9
Glycogen storage disease type II	AR	Profound	*GAA*	15	1.14%	88.1
Hemophilia A	X-LR	Severe	*F8*	3	0.23%	440.3
Duchenne muscular dystrophy	X-LR	Severe	*DMD*	1	0.08%	1321.0
Hemophilia B	X-LR	Severe	*F9*	0	0.00%	NA

AR: autosomal recessive; X-LR: X-linked recessive.

**Table 2:** Estimated gene carrier rates of eight autosomal recessive conditions.

Conditions	Gene	Estimated GCRs
Alpha thalassemia	*HBA1/HBA2*	7.76%
Beta thalassemia	*HBB*	2.25%
Autosomal recessive deafness 1A	*GJB2*	1.66%
Autosomal recessive deafness 4 with enlarged vestibular aqueduct	*SLC26A4*	2.03%
Phenylketonuria	*PAH*	2.18%
Wilson’s disease	*ATP7B*	1.65%
Spinal muscular atrophy	*SMN1*	1.59%
Glycogen Storage Disease type II	*GAA*	1.13%

GCRs: Gene carrier rates.

**Table 3:** Reasons for declining sequential screening.

Reasons	Percentage	Carrier Conditions of the Pregnant Women (No. of Subjects)
Partner has been tested before	48.30% (71/147)	Thalassemias (69), DFNB4 (1), both alpha thalassemia and spinal muscular atrophy (1)
Can accept an affected baby	10.20% (15/147)	Phenylketonuria (2), DFNB1/4 (13)
No risk of Hb Bart syndrome	25.85% (38/147)	Alpha 3.7 (27), alpha 4.2 (11)
Others (high cost, no interest, consideration of other evaluations, etc.)	8.16% (12/147)	Alpha thalassemia (2), DFNB1(2), phenylketonuria (4), Wilson’s disease (2), glycogen storage disease type II (2)
NA	7.48% (11/147)	Thalassemias (2), DFNB4 (2), phenylketonuria (2), Wilson’s disease (1), spinal muscular atrophy (1), both Wilson’s disease and alpha thalassemia (1), glycogen storage disease type II (2)

NA: Not available; DFNB1: autosomal recessive deafness 1A; DFNB4: autosomal recessive deafness 4 with enlarged vestibular aqueduct.

**Table 4:** At-risk couples identified in this cohort.

Conditions	Inheritance Mode	Gene	Variant Type of Mother	Variant Type of Father	Prenatal Diagnosis	Affected Pregnancy	Decision	Pregnancy Outcome
Alpha thalassemia	AR	*HBA1/HBA2*	SEA	SEA	No	NA	Keep pregnancy	NA
Beta thalassemia	AR	*HBB*	c.126_129delCTTT	c.79G>A	No	NA	Keep pregnancy	Live birth
Phenylketonuria	AR	*PAH*	c.158G>A	c.158G>A	Yes	Yes	Keep pregnancy	Live birth
Spinal muscular atrophy	AR	*SMN1*	Exon 7 deletion	Exon 7 deletion	Yes	Yes	TOP	TOP
Hemophilia A	X-LR	*F8*	c.3637delA	NA	Yes	Yes	TOP	TOP
Hemophilia A	X-LR	*F8*	c.1569G>T	NA	No	NA	Keep pregnancy	Live birth
Hemophilia A*	X-LR	*F8*	Intron 22 inversion	NA	No	No	Keep pregnancy	Live birth
Dystrophinopathies	X-LR	*DMD*	EX49 DEL	NA	Yes	No	Keep pregnancy	NA

* The fetus of this couple is a female according to the non-invasive prenatal test (NIPT) report, so she would not be affected with hemophilia A. AR: autosomal recessive; X-LR: X-linked recessive; NA: not available due to loss of follow up; TOP: termination of pregnancy.

## Data Availability

The data are not publicly available because public access was not consented from the subjects in the study, but could be available upon reasonable request from the corresponding author.

## Supplementary Materials

The following are available online at https://www.mdpi.com/article/10.3390/genes12040496/s1, Table S1; Variants detected in our cohort; Table S2: Comparison of sequential testing compliance rates between women carrying one condition and two conditions; Table S3: Comparison of sequential testing compliance rates among women carrying with different severity of conditions.
